# Genuine Gonococcic Stomatitis in Adults

**Published:** 1915-05

**Authors:** 


**Genuine Gonococcic Stomatitis in Adults.** Zilz, Oesterreich.-Ungar. Vierteljahrsschrift für Zahnheilkunde. Vol. 27, No. 2. A case is mentioned in a waitress aged 21, occurring from obvious method of infection and lasting 6 weeks. The literature of this condition is reviewed at length.

